# [Retracted] Erastin sensitizes glioblastoma cells to temozolomide by restraining xCT and cystathionine-γ-lyase function

**DOI:** 10.3892/or.2026.9094

**Published:** 2026-03-09

**Authors:** Liangyu Chen, Xinxing Li, Libo Liu, Bo Yu, Yixue Xue, Yunhui Liu

Oncol Rep 33: 1465–1474, 2015; DOI: 10.3892/or.2015.3712

Following the publication of the above paper, it was drawn to the Editor's attention by a concerned reader that, in addition to the apparent duplication of a pair of protein blots between Fig. 3B and E on p. 1469, some of the western blot data featured in the same figure were strikingly similar to data that had already been submitted for publication to the journal *Molecular and Cellular Biology* in a paper written by different authors at different research institutes.

Owing to the fact that the abovementioned data had already been submitted for publication prior to the receipt of this article at *Oncology Reports*, the Editor has decided that this paper should be retracted from the Journal. The authors were asked for an explanation to account for these concerns, but the Editorial Office did not receive a reply. The Editor apologizes to the readership for any inconvenience caused.

